# 5-HT_2C_ Receptors Localize to Dopamine and GABA Neurons in the Rat Mesoaccumbens Pathway

**DOI:** 10.1371/journal.pone.0020508

**Published:** 2011-06-07

**Authors:** Marcy J. Bubar, Sonja J. Stutz, Kathryn A. Cunningham

**Affiliations:** Department of Pharmacology and Toxicology and Center for Addiction Research, University of Texas Medical Branch, Galveston, Texas, United States of America; Sapienza University of Rome, Italy

## Abstract

The serotonin 5-HT_2C_ receptor (5-HT_2C_R) is localized to the limbic-corticostriatal circuit, which plays an integral role in mediating attention, motivation, cognition, and reward processes. The 5-HT_2C_R is linked to modulation of mesoaccumbens dopamine neurotransmission via an activation of γ-aminobutyric acid (GABA) neurons in the ventral tegmental area (VTA). However, we recently demonstrated the expression of the 5-HT_2C_R within dopamine VTA neurons suggesting the possibility of a direct influence of the 5-HT_2C_R upon mesoaccumbens dopamine output. Here, we employed double-label fluorescence immunochemistry with the synthetic enzymes for dopamine (tyrosine hydroxylase; TH) and GABA (glutamic acid decarboxylase isoform 67; GAD-67) and retrograde tract tracing with FluoroGold (FG) to uncover whether dopamine and GABA VTA neurons that possess 5-HT_2C_R innervate the nucleus accumbens (NAc). The highest numbers of FG-labeled cells were detected in the middle versus rostral and caudal levels of the VTA, and included a subset of TH- and GAD-67 immunoreactive cells, of which >50% also contained 5-HT_2C_R immunoreactivity. Thus, we demonstrate for the first time that the 5-HT_2C_R colocalizes in DA and GABA VTA neurons which project to the NAc, describe in detail the distribution of NAc-projecting GABA VTA neurons, and identify the colocalization of TH and GAD-67 in the *same* NAc-projecting VTA neurons. These data suggest that the 5-HT_2C_R may exert direct influence upon both dopamine and GABA VTA output to the NAc. Further, the indication that a proportion of NAc-projecting VTA neurons synthesize and potentially release both dopamine and GABA adds intriguing complexity to the framework of the VTA and its postulated neuroanatomical roles.

## Introduction

The ventral tegmental area (VTA; A10) is the site of origin of dopamine neurons that send efferent projections to a variety of areas throughout the brain [1]–[3], a large proportion of which project to the nucleus accumbens (NAc) [3]. This dopamine mesoaccumbens projection plays an integral role in mediating attention, motivation, cognition, and reward processes [4], and has also been implicated in the actions of drugs of abuse such as cocaine [5], [6].

The serotonin 5-HT_2C_ receptor (5-HT_2C_R), one of thirteen G-protein coupled serotonin receptor subtypes [7], has been described to exert an overall inhibitory influence over the function of the dopamine mesoaccumbens pathway (for reviews, see [5], [8], [9]). Given that stimulation of the 5-HT_2C_R is expected to evoke neuronal depolarization [10], the 5-HT_2C_R-induced inhibition of basal firing VTA dopamine neurons and release of dopamine in the NAc [11]–[13] is historically thought to be mediated via depolarization of inhibitory γ-aminobutyric acid (GABA) neurons that synapse onto dopamine cell bodies in the VTA [11]–[13]. Interestingly, the 5-HT_2C_R has also been identified within neurons labeled with the dopamine synthetic enzyme tyrosine hydroxylase (TH) [14]. Our previous study demonstrated 5-HT_2C_R colocalization within dopamine neurons [14] in VTA subnuclei that serve as the origin of the densest efferent projections from the VTA to the NAc [3]. Thus, 5-HT_2C_R modulation of the output of the mesoaccumbens pathway is multifaceted at both the level of the dopamine and GABA neurons of the VTA. Further, there is a high potential for the 5-HT_2C_R to influence VTA output to the NAc and, likewise, the numerous physiological and psychological processes mediated by this pathway.

The goal of the present study was to examine the distribution of 5-HT_2C_R in dopamine and GABA VTA neurons that project to the NAc shell employing double-label fluorescence immunohistochemistry and retrograde tracing with the compound FluoroGold (FG). Double-label fluorescence immunohistochemistry for TH+5-HT_2C_R and glutamic acid decarboxylase isoform 67 (GAD-67)+5-HT_2C_R was performed on VTA sections from brains of male Sprague-Dawley rats that received a unilateral injection of FG into the NAc shell [15]. An assessment of cells labeled for FG+TH+5-HT_2C_R indicates the localization of 5-HT_2C_R on dopamine neurons that project to the NAc, while colocalization of FG+GAD-67+5-HT_2C_R reflects the presence of 5-HT_2C_R on GABA projection neurons to the NAc. Additionally, given the recent evidence of coexpression of TH- and GAD-IR in the VTA [16], we also examined the presence of cells labeled for FG+TH+GAD-67 to identify mesoaccumbens neurons that have the potential to synthesize both neurotransmitters.

## Methods

### Ethics Statement

All experiments conformed to the NIH Guide for the Care and Use of Laboratory Animals and were approved by the University of Texas Medical Branch Animal Care and Use Committee protocol 88-03-039.

### Animals and Retrograde Tracing

Naïve male Sprague-Dawley rats (n = 6; virus antibody-free; Harlan, Houston,TX) aged 8–10 weeks and weighing 250–300 g were used in these studies. All rats were maintained in the colony room for a minimum of seven days after arrival, where food and water was available *ad libitum.* Rats were deeply anesthetized using an intramuscular injection of 43 mg/kg of ketamine, 8.6 mg/kg of xylazine and 1.5 mg/kg of acepromazine in 0.9% saline. With the upper incisor bar of a stereotaxic instrument positioned at −3.3 mm below the interaural line and using the intersection of bregma and longitudinal sutures as the origin, FluoroGold (FG; Fluorochrome, Englewood, CO) was unilaterally injected into the NAc shell at 1.4 mm anterior to bregma, 0.75 mm lateral to the midline, and 8.0 mm ventral to the skull surface [17]. A 1–2% FG solution (dissolved in 0.9% saline) was injected through a 5 µL Hamilton syringe fitted with a 26 gauge, blunt-tip (style 3) needle (part #7768-02; Hamilton Company, Reno, NV) mounted onto a stereotaxic frame. The injection was driven by a Micro4 Controller (World Precision Instruments, Sarasota, FL) at a rate of 10 nL/min over 10 min for a total volume of 100 nL. The syringe was left in place for an additional 10 min to allow for full diffusion of the solution out of the syringe. The needle was then reversed slowly to minimize leakage of FG into the infusion track. Following infusion, rats received a single injection of sodium ampicillin after surgery and recovered for one week, during which the rats were handled and weighed daily.

Seven days following FG infusion, rats were deeply anesthetized with pentobarbital (100 mg/kg, IP) then perfused transcardially with phosphate buffered saline (PBS) followed by ∼500 ml of 3% paraformaldehyde in PBS. Brains were then removed, post-fixed for 2 h in 3% paraformaldehyde in PBS, then cryoprotected in 30% sucrose for 2 days at 4°C. Using crushed dry ice, the brains were rapidly frozen and stored at –80°C until sectioning. Coronal sections containing the NAc (30 µm; bregma +0.70 mm through +2.0 mm) and VTA (20 µm; bregma −4.8 mm through −6.5 mm) were taken from all brains using a cryostat (Leica CM 1850 at 20°C; Leica Microsystems, Bannockburn, IL) according to the atlas of Paxinos and Watson [17]. Free floating sections were processed as described below.

### Antibodies

The primary antibodies employed in the present studies include, the goat polyclonal anti-5-HT_2C_R antibody [SR-2C (N-19); Santa Cruz Biotechnology, Santa Cruz, CA], the mouse monoclonal anti-TH antibody (#22941; Immunostar, Hudson, WI), and the rabbit polyclonal anti-GAD 67 antibody (GAD-67; H101; Santa Cruz Biotechnology). Additional details are summarized in **Table 1**. Fluorescent-conjugated secondary antibodies (1∶2000) obtained from Molecular Probes were utilized to visualize primary antibody staining: Alexa Fluor 488 donkey anti-goat, Alexa Fluor 555 donkey anti-rabbit, Alexa Fluor 555 donkey anti-mouse. The Alexa Fluor 488 antibody has an excitation/emission maxima of 491/515 and appears green, while the Alexa Fluor 555 antibodies have an excitation/emission maxima of 573/596 and appear red. Double-label immunohistochemistry experiments for 5-HT_2C_R plus TH and 5-HT_2C_R plus GAD-67 were performed, as well as immunohistochemistry in the presence or absence of each antibody alone, on the FG-injected rat brains to determine the distribution of 5-HT_2C_R on dopamine and GABA cells that project to the NAc, respectively. To examine the possibility of co-labeling of TH and GAD-67 in the same cells, we also conducted double-label immunohistochemistry on FG-labeled cells in the VTA sections for TH plus GAD-67 using Alexa Fluor 488 goat anti-mouse, and Alexa Fluor 555 goat anti-rabbit secondary antibodies, respectively, in one brain.

**Table 1:** Primary antibodies employed in the experiments.

Antibody	SR-2C (N-19)	Tyrosine Hydroxylase	GAD-67 (H101)
**Immunogen**	Human 5-HT_2C_R N-terminus (27-45)	Tyrosine hydroxylase purified from rat PC12 cells	Human GAD-67 N-terminus (1-101)
**Manufacturer**	Santa Cruz Biotechnology (Santa Cruz, CA)	Immunostar (Hudson, WI)	Santa Cruz Biotechnology (Santa Cruz, CA)
**Catalog/lot #**	sc-15081/G102	22941/136932	sc-5602/B211
**Species**	Goat polyclonal	Mouse monoclonal	Rabbit polyclonal
**Dilution**	1∶100	1∶3000	1∶150

### Antibody Characterization

#### SR-2C (N-19)

This anti-5-HT_2C_R antibody (sc-15081, Santa Cruz Biotechnology) recognizes amino acids 27–45 of the 5-HT_2C_R, as determined by MALDI-TOF/TOF mass spectrometry (Applied Biosystems 4800 TOF/TOF) of the blocking peptide (sc-15081-P, Santa Cruz) and confirmed by a representative from Santa Cruz (personal communication). The robust and reproducible pattern of immunostaining previously demonstrated with the N-19 antibody in mouse and rat brain [14], [18], [19] was consistent with that documented using a laboratory-generated 5-HT_2C_R antibody designed against a different epitope [20]. Further, immunostaining for the N-19 antibody was eliminated in transgenic mice lacking the 5-HT_2C_R gene relative to wild type mice [18]. In addition, this anti-5-HT_2C_R antibody did not stain parental Chinese hamster Ovary (CHO) K1 cells (which do not express the 5-HT_2C_R mRNA) [21], [22] or CHO cells that stably express the closely-related 5-HT_2A_R [18]. Western blot analyses of cortical rat brain tissue revealed multiple immunoreactive bands within the predicted molecular weight range of the 5-HT_2C_R which were eliminated via peptide neutralization (sc-15081-P, Santa Cruz Biotechnology) [19], [22] and absent in peripheral organs that do not express the 5-HT_2C_R transcript (e.g., kidney, lung) [22]. The anti-5-HT_2C_R-immunoreactive bands were also present in immunoblots of CHO cells that stably express the 5-HT_2C_R, but were not observed in immunoblots of parental CHO K1 cells which lack the 5-HT_2C_R [22]. In the CHO cells stably expressing the 5-HT_2C_R, the blocking peptide (sc-15081-P, Santa Cruz Biotechnology) effectively blocked the immunoreactive signal produced by the N19 antibody (sc-15081, Santa Cruz); this was demonstrated using both standard fluorescence immunocytochemistry as well as a fixed-cell quantitative plate immunoassay [23] (unpublished observations).

#### TH

This anti-TH antibody (#22941, Immunostar), raised against TH purified from rat PC12 cells, produces patterns of immunoreactive staining in the VTA [14], [24] similar to that reported using other anti-TH antibodies [1], [25], [26]. Anti-TH immunostaining was eliminated by preabsorption of the antibody with a high concentration of TH [27], as well as following toxin-induced lesions of catecholamine neurons [28]. The antibody was characterized in Western blot analyses by Immunostar (Hudson, WI), and was shown to recognize the expected single band of 60 kD in cell extracts of HEK293 cells transiently transfected with cDNA of human TH isoform without cross-reactivity with dihydropterdine reductase, dopamine- β- hydroxylase, phenylethanolamine-N-methyltransferase, phenylalanine hydroxylase, or tryptophan hydroxylase or related enzymes.

#### GAD-67 (H101)

This anti-GAD 67 antibody (sc-5602, Santa Cruz Biotechnology), raised against amino acids 1–101 of the enzyme, detects similar numbers of cells as detected using an anti-GABA antibody in immunohistochemical analyses [29]. Validation studies conducted by Santa Cruz Biotechnology revealed that the anti-GAD-67 antibody detected the decrease in GAD-67 protein expression induced by GAD-67 siRNA transfection in HeLa cells (C. Maraviglia, Santa Cruz Biotechnology, personal communication). In addition, using this antibody, a decrease in GAD-67-IR staining in the VTA was observed in parallel with a decrease in GAD-67 mRNA expression following neurotoxic lesion of VTA GABA neurons [24]. Furthermore, lipopolysaccharide-induced enhancement of the number of GAD-67-expressing cells was detected using this antibody in either Western blot or immunohistochemical analysis, an observation that paralleled the increase in GABA-positive cells induced by lipopolysaccharide [29].

### Immunohistochemistry

Using established techniques [14], [18], [19], free floating rat brain sections were washed using an orbital shaker in PBS (2×10 min), then incubated in 20 mM sodium acetate (1×15 min, RT), and washed again (3×10 min) with PBS. The sections were then incubated in a blocking serum (1.5% normal donkey or goat serum in PBS) for one hour (RT). The blocking serum was aspirated, and the sections were then incubated with primary antibodies (see **Table 1**) diluted in 1.5% normal donkey or goat serum for 44 h on an orbital shaker at 4°C. The sections were then washed with PBS (6×6 min) on an orbital shaker and incubated with the secondary antibodies diluted in 1.5% normal donkey or goat serum for 1 h at room temperature (protected from light). The sections were washed with PBS (3×10 min) and mounted using a 0.1% Drefts solution onto slides previously coated with gelatin chrom alum. The slides were then coverslipped using Vectashield fluorescent mounting medium (Vector Laboratories), and stored protected from light at 4°C until viewing.

### Image Analysis

Digital images were captured from brain sections using an Olympus BX51 fluorescent microscope equipped with a Hamamatsu digital camera (Hamamatsu, Bridgewater, NJ) interfaced to a personal computer and were adjusted using Simple PCI software (version 5.1, Compix Inc., Imaging Systems, Cranberry Township, PA). A 10x, 20x or 40x objective was used to capture all photomicrographs for final magnification of 200x, 400x or 800x, respectively. Green fluorescence emitted by the Alexa Fluor 488 antibodies was visualized using a yellow GFP filter set (#41017; Chroma Technology Corporation, Rockingham, VT), while the red fluorescence emitted by the Alexa Fluor 555 antibodies was visualized using a narrow band green excitation filter set (U-MNG2, Olympus). In addition, FG staining was visualized using a wideband ultraviolet excitation filter set (U-MWU2, Olympus).

Three images of the same viewing area were captured for each section, one for each filter set, and then resultant images were merged. Antibody-specific IR was defined as IR that was visible in tissue sections labeled with the appropriate primary-secondary antibody combination, but was not detectable in control sections containing either the primary or secondary antibody alone. The brightness and contrast of each image was adjusted to eliminate background staining; the settings differed for each antibody utilized and were based upon the level of background staining in the control sections (no primary antibody) that were processed simultaneously for each brain analyzed.

Rostro-caudal patterns of FG labeling and 5-HT_2C_R-, TH-, and GAD-67-IR were analyzed at rostral (bregma −5.00 to −5.40 mm), middle (bregma −5.50 to −5.90 mm), and caudal (bregma −6.00 to –6.40 mm) levels of the VTA [17]. Three to four FG+TH+5-HT_2C_R-labeled sections and adjacent (when possible) FG+GAD-67+5-HT_2C_R-labeled sections per each rostro-caudal level were examined from each rat identified as having an accurately placed unilateral injection of FG into the NAc shell (n = 3 of 6). For each section, a composite photomicrograph comprised of 20–30 individual images captured using the 20x objective was assembled to visualize the entire VTA. Labeled cells were identified in each section and manually counted using the “Count” feature of the Adobe Photoshop CS4 Extended software (Adobe Systems Incoprorated, San Jose, CA). First, with only the blue channel visible, all FG-labeled cells were counted and marked with a blue square. Next, using blue and red channels, each FG+TH- or FG+GAD-67-labeled cell (depending on the staining for the particular section) was counted and marked with a red square. Then, using the blue and green channels and with the TH/GAD-67 count group invisible, all FG+5-HT_2C_R-labeled cells were counted and marked with a green square. Finally, using the blue channel only and with all three count groups visible, each cell that contained blue, red and green squares were marked and counted as being labeled for FG+TH+5-HT_2C_R or FG+GAD-67+5-HT_2C_R (depending on the staining combinations for the particular section); each cell that contained blue and red squares only were marked and counted as being labeled for FG+TH or FG+GAD-67; and each cell that contained blue and green squares were marked and counted as being labeled for FG+5-HT_2C_R. The total number of cells labeled for FG, FG+TH or FG+GAD-67, FG+5-HT_2C_R, and FG+TH+5-HT_2C_R or FG+GAD-67+5-HT_2C_R were recorded for each section. [14], [30].

The total number FG-labeled cells for each section was averaged (± SEM) for rostral, mid and caudal levels. Likewise, the total number of TH-IR cells (sum of FG+TH plus FG+TH+5-HT_2C_R), GAD-67-IR cells (sum of FG+GAD-67 plus FG+GAD-67+5-HT_2C_R) and 5-HT_2C_R-IR cells (sum of FG+5-HT_2C_R plus FG+TH+5-HT_2C_R, or FG+5-HT_2C_R plus FG+GAD-67+5-HT_2C_R) were calculated for each appropriately-labeled section and then averaged (± SEM) for rostral, mid and caudal levels. Subsequently, the percentage of FG+TH or FG+GAD-67-labeled cells was determined by dividing the number of total FG+TH- or FG+GAD-67-labeled cells, respectively, by the total number of FG-labeled cells in that section. The resultant values were averaged (mean ± SEM) for rostral, middle, and caudal levels. In addition, the percentage of FG+TH- or FG+GAD-67-labeled cells that also contained 5-HT_2C_R-IR was determined for each section by dividing the number of FG+TH+5-HT_2C_R- or FG+GAD-67+5-HT_2C_R-labeled cells by the total FG+TH- or FG+GAD-67-labeled cells, respectively. A one-way analysis of variance (ANOVA) was used to determine significant differences in: (1) the number of FG-, FG+TH-, FG+GAD-67-, or FG+5-HT_2C_R-labeled cells across the three rostro-caudal levels of the VTA: (2) the percentage of total FG+TH-, FG+GAD-67-, FG+TH+5-HT_2C_R-, or FG+GAD-67+5-HT_2C_R-labeled cells detected in the rostral, middle and caudal levels of the VTA [31]. Significant effects were followed with post hoc analyses using the Student Newman Keuls procedure [32].

To verify the observation of colocalization of FG+TH+5-HT_2C_R and FG+TH+GAD, brain sections from one additional animal with a FG injection correctly placed in the NAc shell were processed, as described above, and viewed in the BSL2-Advanced Optical Microscopy Core of the UTMB Galveston National Laboratory using an Olympus Fluoview 1000 UV laser scanning confocal microscope mounted on an upright BX61 microscope (Olympus America Inc., Center Valley, PA). Images were captured using a 60x 1.2NA water immersion lens (Olympus). Sets of images were captured with the Fluoview Workstation software (Olympus) using three different fluorescent filter sets to detect the individual fluorophores and the resultant images were overlaid. A series of consecutive image sets were captured at approximately 0.5 µm intervals through the depth of the 20 µm section of the VTA.

## Results

### Retrograde Labeling with FG

**Figure 1:**
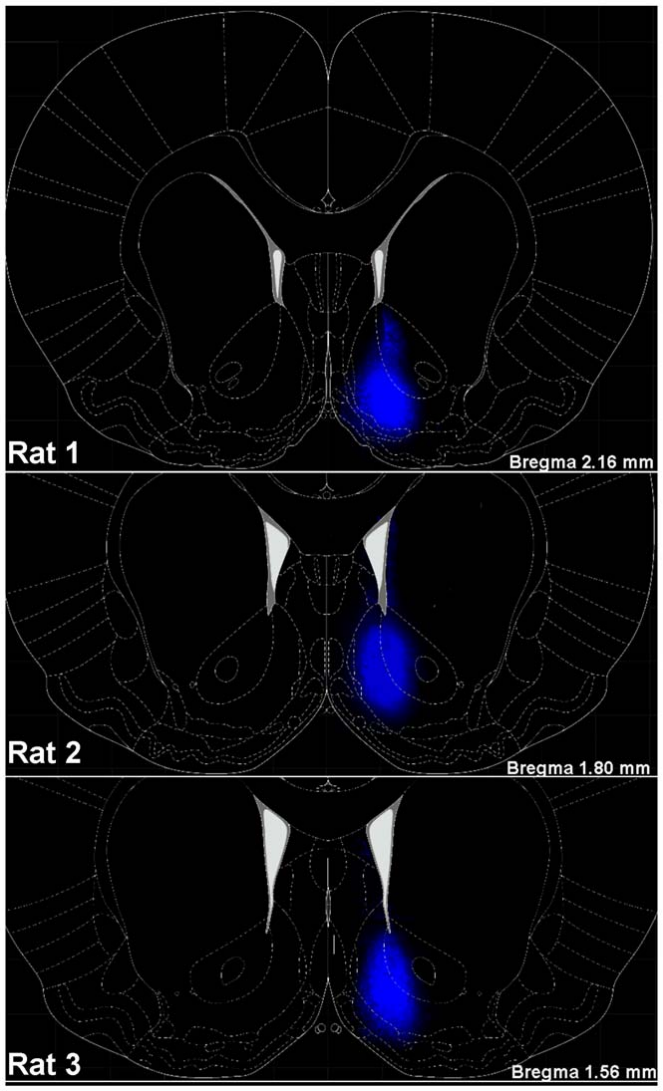
FluoroGold staining at injection site in the NAc. Schematic diagrams depicting coronal sections of the NAc shell and surrounding brain areas at bregma +2.16, +1.80 and +1.56 mm [17] overlaid on top of a representative composite photomicrographs depicting the FG injection sites (100 nL; 1–2% FG) for the three animals with injections correctly placed in the NAc shell.

Of the six animals that received FG injections for semi-quantitative analyses of immunolocalization, three animals had unilateral injections that were correctly placed in the NAc shell (**Fig. 1**). The locations of the three NAc shell injections and the extent of lateral diffusion of FG from the injection site to adjacent brain areas are portrayed in **Fig. 1**. The FG immunofluorescence was primarily confined to the NAc shell with some diffusion into the NAc core, ventral pallidum and islands of calleja/olfactory tubercle (**Fig. 1**). Of the remaining three animals, two of the injections were slightly lateral, with diffusion of FG limited to the NAc core, and the final injection was medial, placed in the septum/diagonal band (data not shown). Only the three brains with injections correctly placed in the NAc shell were processed for a semi-quantitative analysis of double-label fluorescence immunohistochemistry. One additional animal that received a unilateral FG injection correctly placed in the NAc shell was utilized for confocal microscopy analysis (data not shown).

**Figure 2:**
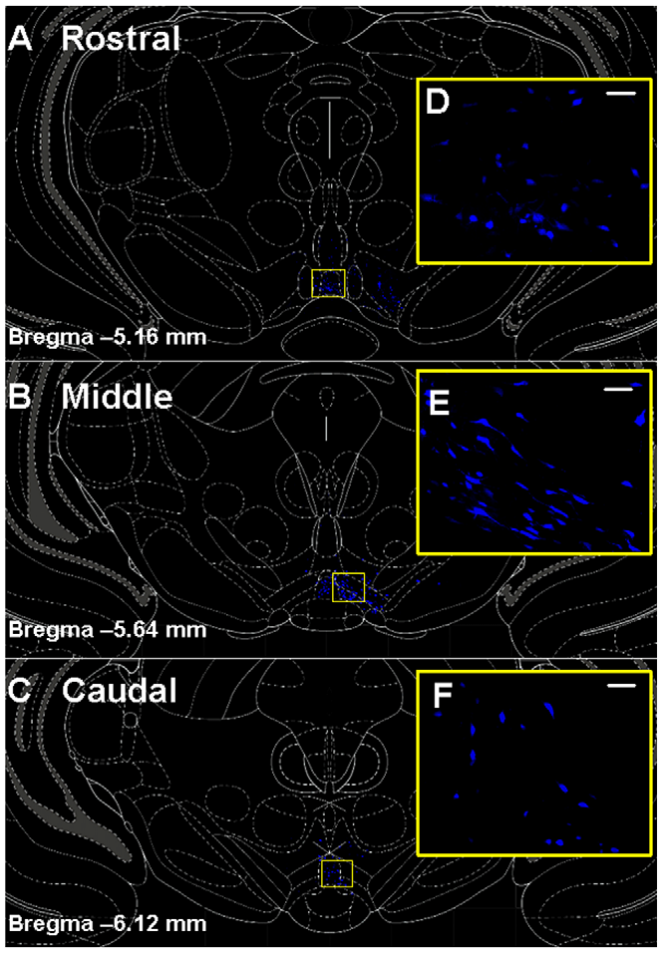
FluoroGold-labeled cells in the VTA following NAc FG infusion. Schematic diagrams depicting coronal sections of the rostral [bregma −5.16 mm; A], middle [bregma −5.64 mm; B], and caudal [bregma −6.12 mm; C,] levels of the VTA [17] and surrounding brain areas overlaid on top of representative composite photomicrographs from Rat #3 (see Fig. 1) displaying FG (blue) labeling in the VTA one week following infusion of FG into the NAc shell. [D, E, F] Higher power magnification of yellow boxed regions in A, B, and C, respectively. Scale bars  = 30 µm.

**Figure 3:**
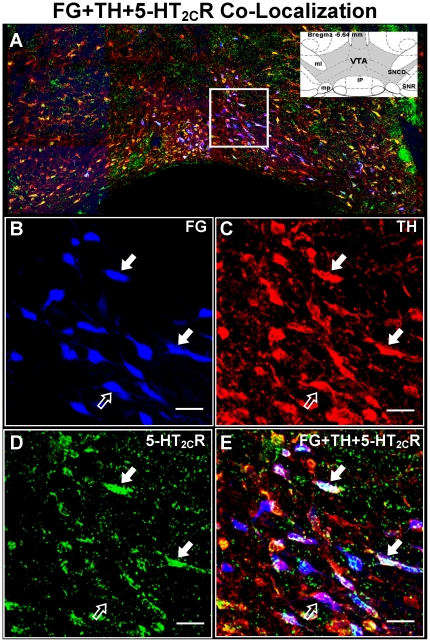
Colocalization of TH and 5-HT_2C_R immuonoreactivity with FG-labeled cells in the VTA. [A] Representative composite photomicrograph of the middle level of the VTA displaying the overlay of FG (blue), TH-IR (red) and 5-HT_2C_R-IR (green). Inset displays the schematic diagram of the middle VTA (shaded area) and surrounding brain areas [interpeduncular nucleus (IP); medial laminiscus (ml); mammillary peduncle (mp); substantia nigra pars compacta, dorsal tier (SNCD); substantial nigra reticulata (SNR)] at bregma −5.64 mm [17]. High magnification images of the boxed region in panel A depict FG labeling [blue; B], TH-IR [red, C], and 5-HT_2C_R-IR [green, D], as well as the overlay of images in B, C and D to demonstrate colocalization [E]. Filled arrows (**
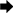
**) indicate cells triple-labeled for FG+TH+5-HT_2C_R, while open arrows (**
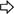
**) indicate a cell double-labeled for FG+TH; Scale bars  = 20 µm. Note: Portions of IP nucleus present in the composite photomicrograph in panel A were removed from the image prior to incorporation into the figure.

**Figure 4:**
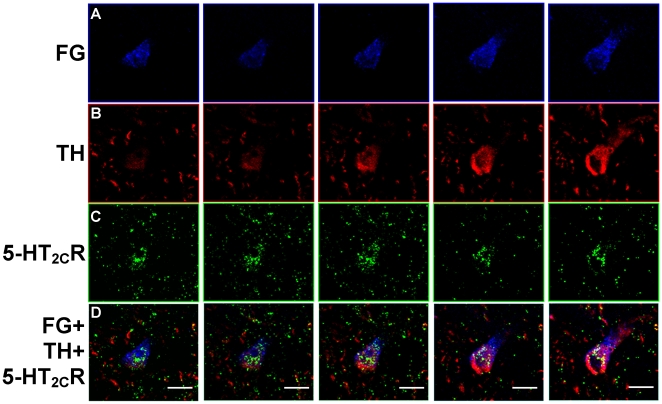
Colocalization of FG, TH and 5-HT_2C_R in the VTA. Images display FG- [blue, A], TH- [red, B] and 5-HT_2C_R-labeling [green, C] in series of five sequential photomicrographs (from left to right) captured using a confocal microscope in the VTA of a rat injected with FG in the NAc shell. Photomicrographs represent images captured at a distance of 1.0 µm apart through the thickness of the brain section. [D] Overlay of images in A–C shows colocalization of TH and 5-HT_2C_R in a FG-labeled cell in the VTA. Scale bar  = 10 µm.

**Figure 5:**
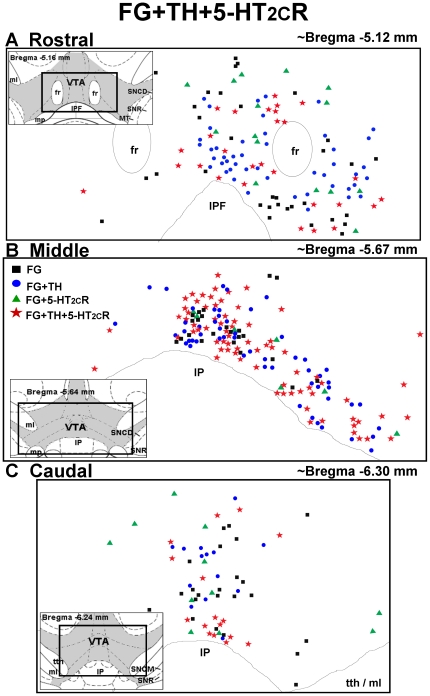
Distribution of FG- TH- and 5-HT_2C_R-labeled cells in the VTA. Schematic representation of the location of cells labeled for FG alone (black squares), FG+TH (blue circles), FG+5-HT_2C_R (green triangles) and FG+TH+5-HT_2C_R-labeled cells (red stars) in the [A] rostral (∼bregma −5.12 mm), [B] middle (∼bregma −5.67 mm), and [C] caudal (∼bregma −6.30 mm) levels of the VTA [17]. Insets display schematic diagrams depicting the location of VTA (shaded) relative to surrounding brain areas [interpeduncular nucleus (IP); interpeduncular fossa (IPF); medial laminiscus (ml); mammillary peduncle (mp); mammillothalamic tract (MT) substantia nigra pars compacta, dorsal tier (SNCD); substantia nigra pars compacta, medial tier (SNCM); substantial nigra reticulata (SNR)] [17]. Data represent the number and distribution of cells counted in one rostral, middle or caudal section from an animal injected with FG in the NAc shell.

Intense labeling for FG was detected in cell bodies and processes in the VTA of rats injected with FG into the NAc (see **Figs. 2**; **3A,B**; **4A**). FG-labeled cells were visible throughout the rostro-caudal extent of the VTA (**Fig. 2**). The vast majority of FG-labeled cells were confined to the side of the brain ipsilateral to the injection site (see **Figs. 2** **,** **3A**). However, a small number of FG-labeled cells were visible in the contralateral VTA, as well as in the ipsilateral substantia nigra (see **Figs. 2** **,** **3A**). **Figure 5** (all symbols combined) provides a schematic illustration of the distribution of all FG-labeled cells detected in a single rostral (**Fig. 5A** **)**, middle (**Fig. 5B** **,)**, and caudal (**Fig. 5C** **)** section of the VTA.

The total number of FG-labeled cells counted in the VTA of each brain (11 sections/brain; n = 3 brains) ranged from 1781–2934. A significant main effect of rostro-caudal level on the distribution of FG cells was observed (F_2,63_ = 34.71; *p*<0.0001; **Table 2**). Significant differences in the average (±SEM) number of FG-labeled cells detected per section were observed between all rostro-caudal levels. The highest number of FG-labeled cells were detected in the middle sections (140.96±7.84), followed by the rostral level (97.00±8.30), and the lowest number of FG-labeled cells detected in the caudal VTA (47.28±6.73) (*p*<0.001; **Table 2**). In the rostral VTA, the FG-labeled cells were most abundant in the ventromedial portions of the VTA subnuclei, with some cells dispersed lateral to the fasciculus retroflexus (fr; see **Fig. 5A**). In the middle level, FG-labeled cells concentrated in the ventral portion of the VTA adjacent to the interpeduncular nucleus (IP; see **Fig. 5B**). In the caudal VTA, FG-labeled cells were concentrated medially, with a few labeled cells scattered laterally (see **Fig. 5C** **)**.

**Table 2:** Total FG-labeled cells containing immunoreactivity for TH, GAD-67, and 5-HT_2C_R.

Level	Rostral	Middle	Caudal	ANOVA
**Bregma Location (mm)** a	**−5.00 to −5.40**	**−5.50 to −5.90**	**−6.00 to −6.40**	
**Number per section:**				
**FG** b	97.00±8.30	140.96±7.84**	47.28±6.73** ∧	F_2,63_ = 34.71 *p*< 0.0001
**FG+TH** b	61.33±5.25	103.42±9.40**	29.67±4.60* ∧	F_2,30_ = 28.13 *p*< 0.0001
**FG+GAD-67** b	47.83±5.98	70.08±5.87*	22.11±5.19* ∧	F_2,30_ = 17.41 *p*< 0.0001
**FG+5-HT_2C_R** c	48.38±3.37	70.88±3.80**	21.94±3.47** ∧	F_2,63_ = 45.40 *p*< 0.0001
**Percentages:**				
**%FG+TH** d	68.31±3.47	75.73±4.42	69.46±6.23	F_2,30_ = 0.89 *p* = 0.42
**%FG+TH+5-HT_2C_R** e	57.17±4.29	58.34±3.73	55.50±6.24	F_2,30_ = 0.10 *p* = 0.91
**%FG+GAD** f	48.59±1.53	49.02±0.87	46.79±2.05	F_2,30_ = 0.64 *p* = 0.53
**%FG+GAD-67+5-HT_2C_R** g	59.66±3.18	55.57±2.22	52.60±4.64	F_2,30_ = 1.24 *p* = 0.30

a Bregma locations according to the brain atlas of Paxinos and Watson (1998).

b Average (± SEM) per section of 12 rostral and middle sections, 9 caudal sections.

c Average (± SEM) number of total FG+5-HT_2C_R-labeled cells per section in 24 rostral and middle, 18 caudal sections.

d Average (± SEM) of FG+TH-labeled cells/total FG cells in 12 rostral and middle, 9 caudal sections.

e Average (± SEM) of FG+TH+5-HT_2C_R-labeled cells/total FG+TH-labeled cells in 12 rostral and middle, 9 caudal sections.

f Average (± SEM) of FG+GAD-67-labeled cells/total FG cells in 12 rostral and middle, 9 caudal sections.

g Average (± SEM) of FG+GAD-67+5-HT_2C_R-labeled cells/total FG/+GAD-67-labeled cells in 12 rostral and mid, 9 caudal sections.

**p<0.001 vs. rostral level;

*p<0.01 vs. rostral level;

∧*p*<0.001 vs. middle level.

### Distribution of FG+TH-labeled cells

Immunofluorescence studies for TH in VTA sections following unilateral injection of FG into the NAc shell revealed that a subset of FG-labeled cells in the VTA contained TH-IR (**Figs. 3**, **4**, **5**). **Figure 3** displays representative photomicrographs demonstrating colocalization of FG+TH in the middle VTA using light microscopy (see 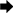 and 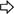 in **Fig. 3B,C**). The presence of TH-IR within FG-labeled cells was confirmed using confocal microscopy (compare **Fig. 4A** with **4B**). A main effect of rostro-caudal level was observed for the distribution of FG+TH cells in the VTA (F_2,30_ = 28.13; *p*<0.0001; **Table 2**). In accordance with that observed for total FG-labeled cells, significant differences in the average (±SEM) number of FG+TH-labeled cells detected per section were observed amongst all the rostro-caudal levels, with middle (103.42±9.40) > rostral (61.33±5.25) > caudal (29.67±4.60) (*p*<0.01; **Table 2**). There was no main effect of rostro-caudal level for the percentage of total FG cells containing TH-IR (∼68-76%; F_2,30_ = 0.89; *p*<0.42; **Table 2**), suggesting that a similar proportion of FG cells were positive for TH-IR across all rostro-caudal levels.

**Figure 5** illustrates the distribution of all FG-labeled cells detected in one rostral (**Fig. 5A**), middle (**Fig. 5B**) and caudal section (**Fig. 5C**) of the VTA of a rat injected with FG into the NAc shell and stained for TH and 5-HT_2C_R IR; the total population of FG-labeled cells that also contained TH-IR include the blue circles, representing cells labeled for FG+TH only, and the red stars, representing cells labeled for FG+TH+5-HT_2C_R (see “Distribution of 5-HT_2C_R in FG+TH-labeled cells,” below). In general, FG+TH-labeled cells were interspersed among all FG-labeled cells detected in the VTA. Of note, virtually all of the FG-labeled cells located in the medial portion of the rostral VTA immediately dorsal to the interpeduncular fossa (IPF) were co-labeled with TH-IR (**Fig. 5A**). In the middle level, almost all of the FG-labeled cells located most ventrally along the interpeduncular nucleus (IP) nucleus were co-labeled with TH (**Fig. 5B**). In the caudal VTA, all FG+TH-labeled cells were concentrated along the midline (**Fig. 5C** **)**.

### Distribution of FG+GAD-67-labeled cells

**Figure 6:**
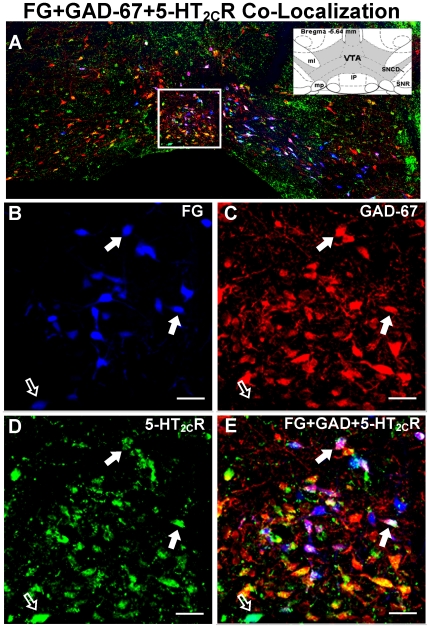
Colocalization of GAD-67 and 5-HT_2C_R immuonoreactivity with FG-labeled cells in the VTA. [**A**] Representative composite photomicrograph of the middle level of the VTA displaying the overlay of FG (blue), GAD-67-IR (red) and 5-HT_2C_R-IR (green). Inset displays the schematic diagram of the middle VTA (shaded area) and surrounding brain areas (see Fig. 3 for abbreviations) at bregma -5.64 mm [17]. High magnification images of the boxed region in panel A depict FG labeling [blue; B], GAD-67-IR [red, C], and 5-HT_2C_R-IR [green, D], as well as the overlay of images in B, C, and D to demonstrate colocalization [E]. Filled arrows (**
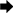
**) indicate cells triple-labeled for FG+GAD-67+5-HT_2C_R cells, while the open arrows (**
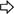
**) indicate a cell double-labeled for FG+5-HT_2C_R; as noted in the text, cells labeled for FG+GAD-67 alone were not often detected in the area represented by the boxed region. Scale bars  = 20 µm. Note: Portions of IP nucleus present in the composite photomicrograph in panel A were removed from the image prior to incorporation into the figure.

**Figure 7:**
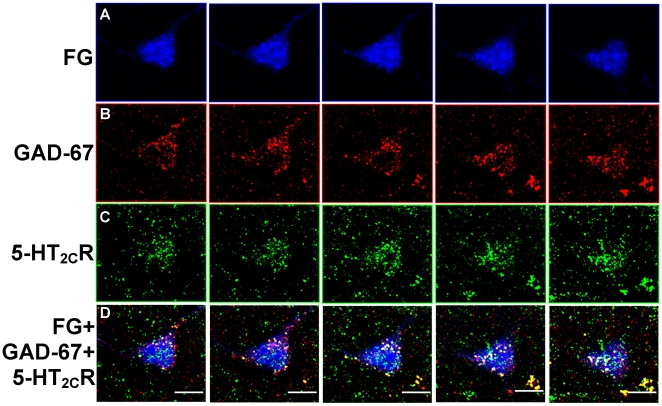
Colocalization of FG, GAD-67 and 5-HT_2C_R in the VTA. Photomicrographs display FG- [blue, A], GAD-67- [red, B] and 5-HT_2C_R-labeling [green, C] in series of five sequential images (from left to right) captured using a confocal microscope in the VTA of a rat injected with FG in the NAc shell. Photomicrographs represent images captured at a distance of 1.0 µm apart through the thickness of the brain section. [D] Overlay of images in A-C shows colocalization of GAD-67- and 5-HT_2C_R-IR in a FG-labeled cell in the VTA. Scale bars  = 10 µm.

A subset of FG cells throughout the VTA contained GAD-67-IR (**Figs. 6** **,** **7** **,** **8**). **Figure 6** displays representative photomicrographs demonstrating colocalization of FG+GAD-67 (see 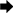 in **Fig. 6B,C**) in the middle VTA using light microscopy. The presence of GAD-67-IR within FG-labeled cells was confirmed using confocal microscopy (compare **Fig. 7A** with **7B**). A main effect of rostro-caudal level was observed for the distribution of FG+GAD-67 cells in the VTA (F_2,30_ = 17.41; *p*<0.0001; **Table 2**). As observed for total FG-labeled cells, significant differences in the average (±SEM) number of FG+GAD-67-labeled cells detected per section were observed amongst all the rostro-caudal levels, across middle (70.08±5.87) > rostral (47.83±5.98) > caudal (22.11±5.19) localization planes (*p*<0.01; **Table 2**). There was no main effect of rostro-caudal level on the percentage of total FG-labeled cells containing GAD-67-IR (∼47-49%; F_2,30_ = 0.23; *p* = 0.79; **Table 2**), suggesting that a similar proportion of FG cells were positive for GAD-67-IR across all rostro-caudal levels.

**Figure 8:**
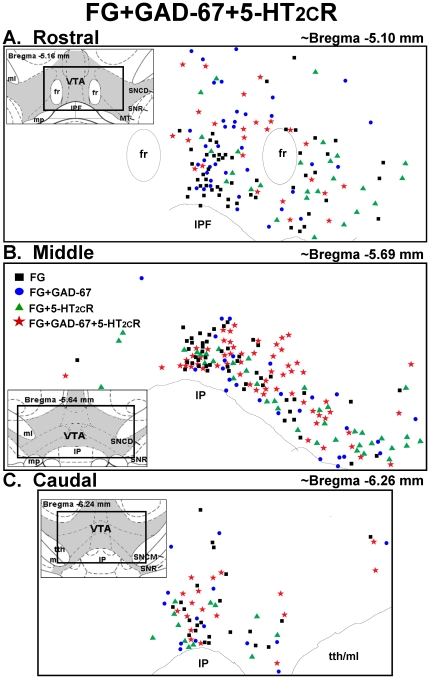
Distribution of FG- GAD-67- and 5-HT_2C_R-labeled cells in the VTA. Schematic representation of the location of cells labeled for FG alone (black squares), FG+GAD-67 (blue circles), FG+5-HT_2C_R (green triangles) and FG+GAD-67+5-HT_2C_R-labeled cells (red stars) in the [A] rostral (∼bregma −5.10 mm), [B] middle (∼bregma −5.69 mm), and [C] caudal (∼bregma −6.26 mm) levels of the VTA [17]. Insets display schematic diagrams depicting the location of VTA (shaded) relative to surrounding brain areas (see Fig. 5 for abbreviations) [17]. Data represent the number and distribution of cells counted in one rostral, middle or caudal section from an animal injected with FG in the NAc shell.

**Figure 8** illustrates the distribution of all FG-labeled cells detected in one rostral (**Fig. 8A**), middle (**Fig. 8B**), and caudal section (**Fig. 8C**) of the VTA of a rat injected with FG into the NAc shell and stained for GAD-67 and 5-HT_2C_R IR; the total population of FG-labeled cells that also contained GAD-67-IR include the blue circles, representing cells labeled for FG+GAD-67 only, and the red stars, representing cells labeled for FG+GAD-67+5-HT_2C_R (see “Distribution of 5-HT_2C_R in FG+GAD-67-labeled cells,” below). In general, FG+GAD-67-labeled cells were interspersed among all FG-labeled cells detected in the rostral, middle and caudal VTA (**Fig 8A-C**). Notably, in the rostral VTA, the majority of FG-labeled cells along the the dorsal end of the midline [dorsal to the fasciculus retroflexus (fr)] contained GAD-67-IR (blue circles and red stars, **Fig. 8A**).

### Distribution of FG+5-HT_2C_R-labeled cells

As described previously (Bubar et al., 2005; Bubar and Cunningham, 2007), 5-HT_2C_R-IR was prominently distributed in the membrane and cytoplasm of both TH-IR and GAD-67-IR perikarya, with potential localization in neuronal processes. 5-HT_2C_R-IR was found to be localized to a subset of FG-labeled cells in the VTA (**Table 2**, **Figs. 3**, 4, 5, 6, 7, 8). **Figures 3** **and** **6** display representative photomicrographs demonstrating colocalization of FG+5-HT_2C_R (see 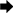 in **Figs. 3B,D** **;** **6B,D**) in the middle VTA using light microscopy. The presence of 5-HT_2C_R-IR within FG-labeled cells was confirmed using confocal microscopy (compare **Fig. 4A** with **4C** and **Fig. 7A** with **7C**). A main effect of rostro-caudal level was observed for the distribution of FG+5-HT_2C_R-labeled cells in the VTA (F_2,63_ = 45.40; *p*<0.0001; **Table 2**). In accordance with all FG-labeled cells, significant differences in the average (±SEM) number of FG+5-HT_2C_R-labeled cells detected per section were observed amongst all rostro-caudal levels of the VTA, with middle (70.88±3.80) > rostral (48.38±3.37) > caudal VTA (21.94±3.47) (*p*<0.001; **Table 2**).

**Figures 5** **and** **8** illustrate the distribution of FG-labeled cells that contain 5-HT_2C_R-IR detected in one rostral (**Fig. 5A** **,** **8A**), middle (**Fig. 5B** **,** **8B**), and caudal section (**Fig. 5C** **,** **8C**) of the VTA of a rat injected with FG into the NAc shell followed by immunohistochemical detection of TH- and 5-HT_2C_R-IR, or GAD-67 and 5-HT_2C_R-IR, respectively. The total population of FG-labeled cells that contained 5-HT_2C_R-IR are represented by the green triangles (FG+5-HT_2C_R-labeled cells, **Figs 5** **and** **8**) and red stars combined (FG+TH+5-HT_2C_R, **Fig. 5**; FG+GAD-67+5-HT_2C_R, **Fig. 8**). The 5-HT_2C_R-IR cells were interspersed among all FG-labeled cells throughout the rostral, middle and caudal levels of the VTA (**Figs. 5A**–**C** **and** **8A**–**C**).

### Distribution of 5-HT_2C_R-IR in FG+TH-labeled cells

Immunoreactivity for the 5-HT_2C_R was observed to be present in the just over half of the FG+TH-labeled cells (**Table 2**; Figs. 3, 4, 5), ranging from ∼56–58% of total FG+TH-labeled cells throughout the VTA. **Figure 3** displays representative photomicrographs demonstrating colocalization of FG+TH+5-HT_2C_R (see 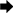 in **Fig. 3B-E**) in the VTA using light microscopy. The presence of both TH- and 5-HT_2C_R-IR in the same FG-labeled cell was confirmed using confocal microscopy (**Fig. 4**). There was no main effect of rostro-caudal level on the percentage of FG+TH-labeled cells containing 5-HT_2C_R-IR (F_2,30_  = 0.10; *p* = 0.91; **Table 2**), suggesting a relatively equal distribution of 5-HT_2C_R-IR in FG+TH labeled cells across rostral-caudal levels of the VTA. As illustrated in **Fig. 5**, no obvious patterns of distribution were noted for FG+TH+5-HT_2C_R-labeled cells, although in the rostral and middle VTA, in particular, the vast majority (>75%) of the 5-HT_2C_R-IR detected was localized to FG cells that also contained TH-IR (**Fig. 5A**–**C**).

### Distribution of 5-HT_2C_R-IR in FG+GAD-67-labeled cells

Immunoreactivity for the 5-HT_2C_R was detected in ∼53–60% of FG+GAD-67-labeled cells identified throughout the VTA (**Table 2**; Figs. 6, 7, 8). **Figure 6** displays representative photomicrographs demonstrating colocalization of FG+GAD-67+5-HT_2C_R (see 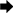 in **Fig. 6B-E**) in the middle VTA using light microscopy. The presence of both GAD-67- and 5-HT_2C_R-IR in the same FG-labeled cell was confirmed using confocal microscopy (**Fig. 7**). There was no main effect of rostro-caudal level on the percentage of FG+GAD-67-labeled cells containing 5-HT_2C_R-IR (F_2,30_ = 1.24; *p* = 0.30; **Table 2**), suggesting a relatively equal distribution of 5-HT_2C_R-IR in FG+GAD-67-labeled cells across rostral-caudal levels of the VTA. As illustrated in **Fig. 8** ****, 5-HT_2C_R-IR was in general equally distributed between FG+GAD-67-labeled cells and those labeled with FG alone (see **
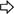
** in **Fig. 6B-E**). Of note, virtually all of the FG+GAD-labeled cells in the medial portion of the middle VTA just dorsal to the IP also contained 5-HT_2C_R-IR (**Figs. 6** **and** **8B**).

### TH and GAD colocalization in the VTA

**Figure 9:**
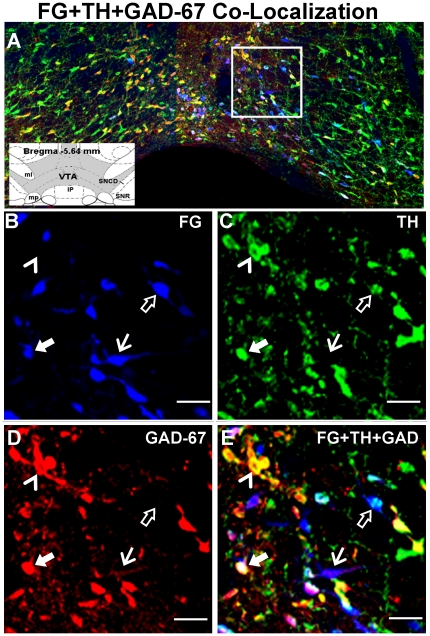
Colocalization of TH and GAD-67 immuonoreactivity with FG-labeled cells in the VTA. [A] Representative composite photomicrograph of the middle level of the VTA displaying the overlay of FG (blue), TH-IR (green) and GAD-67-IR (red). Inset displays the schematic diagram of the middle VTA (shaded area) and surrounding brain areas (see Fig. 3 for abbreviations] at bregma -5.64 mm. [17]. High magnification images of the boxed region in panel A depict FG labeling [blue, B], TH-IR [green, C], and GAD-67-IR [red, D], as well as the overlay of images in B, C, and D to demonstrate colocalization [E]. Filled arrows (**
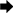
**) indicate a cell triple-labeled for FG+TH+GAD-67, open arrows (**
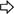
**) indicate a cell double-labeled for FG+TH, solid arrows (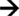) indicate a cell double-labeled for FG+GAD-67, and the arrowheads (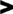) point to a cell double-labeled for TH+GAD-67 in the absence of FG; Scale bars  = 20 µm. Note: Portions of IP nucleus present in the composite photomicrograph in panel A were removed from the image prior to incorporation into the figure.

**Figure 10:**
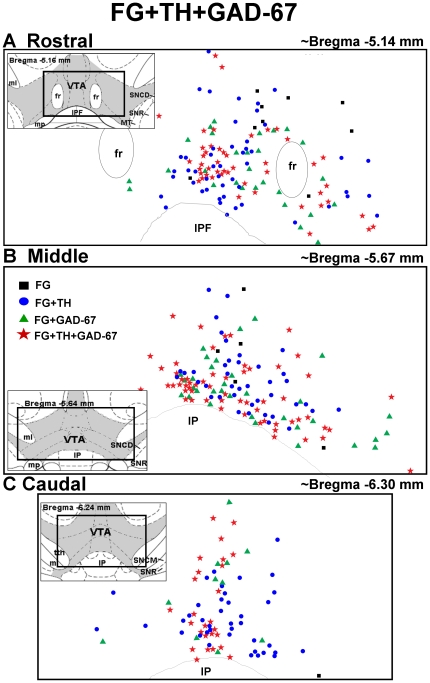
Distribution of FG- TH- and GAD-67-labeled cells in the VTA. Schematic representation of the location of cells labeled for FG alone (black squares), FG+TH (blue circles), FG+GAD-67 (green triangles) and FG+TH+GAD-67-labeled cells (red stars) in the [A] rostral (∼bregma −5.14 mm), [B] middle (∼bregma −5.67 mm), and [C] caudal (∼bregma −6.30 mm) levels of the VTA [17]. Insets display schematic diagrams depicting the location of VTA (shaded) relative to surrounding brain areas (see Fig. 5 for abbreviations) [17]. Data represent the number and distribution of cells counted in one rostral, middle or caudal section from a animal injected with FG in the NAc shell.

The recent report that TH- and GAD-IR co-label in VTA cells [16] and the observation that the sum of the percentage of FG+TH-labeled cells (68-76%) plus FG+GAD-67-labeled cells (47-49%) was slightly greater than 100% for all levels of the VTA (**Table 2** **)** prompted an examination of FG-labeled cells in VTA for immunoreactivity for both TH and GAD-67. **Figure 9** displays representative photomicrographs demonstrating colocalization of FG+TH+GAD-67 the middle VTA using light microscopy. Although a thorough analysis of the distribution was not conducted, we examined one section/level of VTA from one of the NAc FG-injected brains for TH and GAD-67 colocalization (**Fig. 10**). From these few sections, >34% of the total FG-labeled cells appear to contain immunoreactivity for *both* TH and GAD (see 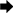, **Fig. 9B-D** **;** red stars, **Fig. 10A-C**), as did a large number of non-FG labeled cells (see 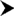, **Fig. 9B-D**). TH+GAD-67 colocalization was present throughout the rostro-caudal extent of the VTA. In general, there was a higher proportion of FG+TH+GAD-67-labeled cells (see 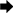, **Fig. 9B-D** **;** red stars, **Fig. 10A-C**) than cells labeled for FG+GAD-67 alone (see 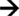, **Fig. 9B-D** **;** green triangles, **Fig. 10A-C**), while the proportion of FG+TH+GAD-67-labeled cells versus FG+TH-labeled cells (see 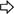, **Fig. 9B-D** **;** blue circles, **Fig. 10A-C**) was more equally distributed. Also of note, there was a relatively high concentration of FG+TH+GAD-67-labeled cells (red stars) with few cells labeled for FG+GAD-67 alone (green triangles) in the ventromedial portions of the rostral and caudal VTA just dorsal to the IP (see **Figs. 9** **and** **10A,C**), indicating that the majority of FG+GAD-labeled cells in these areas also contained TH.

## Discussion

The present study employed a combination of FG retrograde tracing and double-label immunofluorescence techniques to demonstrate for the first time the localization of the 5-HT_2C_R in both dopamine and GABA VTA neurons that project to the NAc, the detailed description of NAc-projecting GABA VTA neurons, and the colocalization of dopamine and GABA neuronal markers in the same NAc-projecting neurons in the VTA. The FG-labeled neurons were most frequently detected in the middle level of the VTA, and a greater proportion of the FG-labeled neurons contained immunoreactivity for TH (68–76%) compared to GAD-67 (47–49%). In addition, across the rostro-caudal extent of the VTA, just over 50% of FG+TH- or FG+GAD-labeled cells also contained immunoreactivity for the 5-HT_2C_R, suggesting that the 5-HT_2C_R has the potential to exert direct influence upon a large population of dopamine and GABA NAc-projecting VTA neurons. Furthermore, the observation that a proportion of FG-labeled (and non-FG-labeled) cells contain immunoreactivity for *both* TH and GAD-67 adds additional complexity to the framework of the VTA and its postulated neuroanatomical roles.

The present study demonstrates that unilateral injection of FG into the NAc shell results in prominent labeling of cells in the ipsilateral VTA, with significantly greater numbers of FG-labeled cells detected in the middle level of the VTA, compared to rostral or caudal levels. Our observation that FG-labeled neurons were generally concentrated in the ventromedial portion of the VTA are in congruence with previous reports describing a mediolateral and inverted dorsoventral topography of projections from the VTA to the NAc [1], [3], [15], [33]–[35], such that projections to the dorsomedial NAc shell, to which our FG infusions were targeted, appear to arise from the ventral and medial areas of the VTA [1], [33]–[35]. The detection of a small number of FG-labeled cells in the dorsolateral and dorsomedial VTA is likely due to the spread of FG into the NAc core and olfactory tubercle/pallidal areas, respectively [1], [33].

Immunofluorescent staining for the synthetic enzymes TH and GAD-67 confirmed that the FG-labeled VTA projection neurons to the NAc are comprised of both dopamine and GABA neurons [3], [33], [36]. Differences in the distribution patterns between NAc-projecting dopamine vs. GABA neurons were very subtle, as the GAD-67-labeled FG neurons were generally interspersed among the larger population of FG+TH-labeled neurons. Here, we also report for the first time the presence of 5-HT_2C_R-IR in >50% of dopamine and GABA neurons that project from the VTA to the NAc [14]. These data confirm our prior results demonstrating colocalization of 5-HT_2C_R-IR with both TH-IR and GAD-IR in the VTA [14], as well as the suggestion by Ji et al [37] that 5-HT_2C_R colocalize to VTA dopamine neurons innervating the NAc. Although our results are in contrast to a previous *in situ* hybridization study in which 5-HT_2C_R mRNA was detected in VTA GABA, but not dopamine, neurons [38], there are several explanations (e.g., differential translational efficiency and protein turnover rates) that are likely to account for the observed dissociation between 5-HT_2C_R mRNA and protein expression in dopamine VTA neurons (see [14] for brief discussion).

The dopamine and GABA neurons that express the 5-HT_2C_R were interspersed throughout the rostral-caudal extent of the VTA, again with only subtle differences in the distribution of these neuronal subpopulations detected. For example, we noted that almost all of the FG-labeled GABA neurons in the medial VTA, just dorsal to the interpeduncular nucleus (IP), appear to contain 5-HT_2C_R. Though not well studied, this area, typically referred to as the interfascicular subnucleus of the VTA, is highly innervated by raphe 5-HT neurons [39] and sends dense projections to the medial NAc shell [1].

The 5-HT_2C_R has been thought historically to exert an inhibitory influence upon mesoaccumbens dopamine neurotransmission via induction of GABA release from axonal collaterals that synapse upon local dopamine neurons within the VTA [12], [13]. The prevailing theory is that 5-HT_2C_R localized to VTA GABA interneurons mediate this effect [11]–[13]. However, the present discovery that the 5-HT_2C_R co-localizes within NAc-projecting GABA neurons provides an additional intriguing site of action for 5-HT_2C_R to modulate mesoaccumbens neurotransmission. While the existence of non-dopaminergic efferent projections from the VTA has been repeatedly documented [2], [3], [33], [40], the nature and role for these remain undisclosed. While it is probable that GABA efferent projections to the NAc form symmetric (inhibitory) synaptic contacts with non-GABAergic dendrites [40], further investigation into the nature of the interactions between VTA GABA projection neurons and these other neuronal systems within the NAc are necessary before we fully appreciate the functional consequences of 5-HT_2C_R in GABA mesoaccumbens neurons.

Our current finding demonstrating the presence of 5-HT_2C_R directly on NAc-projecting mesoaccumbens dopamine neurons does not intuitively fit into the overall concept of 5-HT_2C_R inhibition of mesoaccumbens dopamine neurotransmission, as stimulation of these 5-HT_2C_R would be expected *stimulate* the output of dopamine VTA neurons through an intracellular cascade that results in neuronal depolarization [10]. However, the inhibitory control exerted by the 5-HT_2C_R over the output of dopamine VTA neurons was primarily surmised from research strategies that utilized systemic administration of 5-HT_2C_R ligands [11], [41], [42] or local application of non-selective compounds [12], [43], complicating the interpretation that selective activation of the VTA 5-HT_2C_R is responsible. Conversely, intra-VTA infusion of a selective 5-HT_2C_R agonist, antagonist, or inverse agonist had little to no effect on basal NAc dopamine release [44], [45] while bath application of a 5-HT_2C_R antagonist did not alter the spontaneous activity of VTA dopamine neurons *in vitro* [46]. The present results provide the anatomical data to support these more selective neurochemical analyses, and suggest that under basal conditions, the subpopulations of 5-HT_2C_R within the VTA that act upon dopamine versus GABA neurons counterbalance one another to neutralize the influence that either population alone exerts upon the dopamine mesoaccumbens neurotransmission.

Antagonism of 5-HT_2C_R locally within the VTA, however, dose-dependently reverses the inhibition of dopamine outflow in the NAc induced by systemic administration of a 5-HT_2C_R agonist [44]. Thus, when 5-HT_2C_R mechanisms extrinsic to the VTA are engaged, the inhibitory effects of local VTA 5-HT_2C_R upon phasic DA mesoaccumbens neurotransmission seem to predominate. The mechanisms which underlie this effect are unclear at this time and need to be explored. The 5-HT_2C_R mRNA and protein are present in moderate to high levels throughout the limbic-corticostriatal circuit, including the NAc, prefrontal cortex, cingulate cortex, amygdala, ventral pallidum and hippocampus [20], [47]. Thus it is probable that 5-HT_2C_R localized to one or more of the other nodes within the limbic-corticostriatal circuitry that feed to the VTA are involved. Indeed, selective blockade of 5-HT_2C_R localized to the NAc, also reversed the inhibition of NAc dopamine outflow induced by systemic 5-HT_2C_R agonist administration [44]. Thus, the NAc represents an additional site of action through which the 5-HT_2C_R localized within this complex feedback circuitry may modulate mesoaccumbens DA neurotransmission.

Yet another layer of complexity is brought to light by the present discovery that TH and GAD-67 are co-expressed within a subpopulation of VTA neurons that project to the NAc. Colocalization of GABA or GAD isoforms and synthetic enzymes for catecholamines in the same cell has been reported throughout the brain [48], [49]. Although an early study did not detect colocalization of GABA or GAD and TH in the VTA or the closely related substantia nigra [49], more recent studies have provided immunohistochemical evidence for colocalization of TH and GAD or GABA in neurons of the VTA [16], as well as in a large population of substantia nigra neurons [48]. Our observations are further substantiated by studies demonstrating the colocalization of TH protein with GAD mRNA in the substantia nigra and lateral VTA [50], as well as the presence of TH and GAD mRNA in the same cell as demonstrated via single-cell RT-PCR of cells from the VTA and/or substantia nigra [51], [52]. These provocative data suggest the possibility of simultaneous production, and perhaps release, of the two neurotransmitters from the same VTA neurons with terminals localized in the NAc [16].

The existence of multiple sites of action in the VTA for the 5-HT_2C_R suggests that these receptors play a key role in fine-tuning the activation of mesoaccumbens neurotransmission under stimulated conditions. Likewise, any imbalance in this complex 5-HT_2C_R framework could contribute to dysregulation of mesoaccumbens transmission, possibly contributing to associated physiological and psychological disorders, including depression, schizophrenia and reward-related disorders such as addiction [6], [53], [54]. Or, alternatively the VTA 5-HT_2C_R may represent a critical avenue for reversing/regulating such dysfunction. Indeed, 5-HT_2C_R ligands are under development for the treatment of depression, schizophrenia and drug abuse [5], [55]. However, much investigation is needed to elucidate the functional roles for the multiple sites of action for 5-HT_2C_R in the VTA, and importantly to identify means to selectively target these individual subpopulations of receptors. The data presented here provide the anatomical framework upon which such detailed and selective functional studies can be designed to more fully understand the implications of the multiple sites of action for 5-HT_2C_R in the VTA, and, furthermore, highlight the need for further investigation into the functional role of mesoaccumbens GABA neurons, as well as the potential for co-release of dopamine and GABA from mesoaccumbens neurons.
